# Canagliflozin reduces epicardial fat in patients with type 2 diabetes mellitus

**DOI:** 10.1186/s13098-017-0275-4

**Published:** 2017-10-04

**Authors:** Shusuke Yagi, Yukina Hirata, Takayuki Ise, Kenya Kusunose, Hirotsugu Yamada, Daiju Fukuda, Hotimah Masdan Salim, Gulinu Maimaituxun, Susumu Nishio, Yuriko Takagawa, Saori Hama, Tomomi Matsuura, Koji Yamaguchi, Takeshi Tobiume, Takeshi Soeki, Tetsuzo Wakatsuki, Ken-ichi Aihara, Masashi Akaike, Michio Shimabukuro, Masataka Sata

**Affiliations:** 1 Department of Cardiovascular Medicine, Tokushima University Graduate School of Biomedical Sciences, 3-18-15, Kuramoto-cho, Tokushima, 770-8503 Japan; 2 Department of Community Medicine and Human Resource Development, Tokushima University Graduate School of Biomedical Sciences, Tokushima, Japan; 3 Department of Internal Medicine, Shikoku Central Hospital, Shikokuchuo, Ehime Japan; 4 Ultrasound Examination Center, Tokushima University Hospital, Tokushima, Japan; 5 Department of Cardio-Diabetes Medicine, Tokushima University Graduate School of Biomedical Sciences, Tokushima, Japan; 6 Department of Community Medicine for Diabetes and Metabolic Disorders, Tokushima University Graduate School of Biomedical Sciences, Tokushima, Japan; 7 Department of Medical Education, Tokushima University Graduate School of Biomedical Sciences, Tokushima, Japan; 8 Department of Diabetes, Endocrinology and Metabolism, School of Medicine, Fukushima Medical University, Fukushima, Japan

**Keywords:** Epicardial adipose tissue, SGLT2 inhibitors, Echocardiography

## Abstract

**Background:**

It is unknown whether canagliflozin, a selective sodium glucose co-transporter 2 inhibitor, reduces epicardial adipose tissue (EAT) thickness, which is associated with insulin resistance and is a risk factor for coronary artery disease.

**Methods and results:**

We administered 100 mg of canagliflozin for 6 months to 13 patients with type 2 diabetes mellitus. We evaluated glycemic control, visceral adipose tissue (VAT) area and subcutaneous adipose tissue (SAT) area, and skeletal muscle mass by using impedance methods, and EAT thickness by using echocardiography. Canagliflozin treatment for 6 months decreased hemoglobin A1c level from 7.1 ± 0.5% to 6.7 ± 0.6% (P < 0.05) and decreased EAT thickness from 9.3 ± 2.5 to 7.3 ± 2.0 mm (P < 0.001), along with a trend of decreasing VAT and SAT area. No association was found between any of these changes.

**Conclusion:**

Canagliflozin reduced EAT thickness in patients with type 2 diabetes mellitus independent of its effect on lowering blood glucose, suggesting that canagliflozin may have an effect in preventing cardiovascular events in these patients (UMIN000021327).

**Electronic supplementary material:**

The online version of this article (doi:10.1186/s13098-017-0275-4) contains supplementary material, which is available to authorized users.

## Background

Epicardial adipose tissue (EAT) is associated with various cardiovascular risk factors such as body mass index and area of visceral adipose tissue, as well as with the severity of coronary artery disease (CAD) [1–4]. Insulin resistance is closely associated with the physiology of adipose tissue [5–7]. In addition, it has been reported that various interventions that improve insulin resistance such as diet therapy and exercise also reduce EAT, which implies that insulin resistance is a potential target in reducing EAT and preventing CAD [8].

Selective sodium glucose co-transporter 2 (SGLT2) inhibitors improve glucose metabolism by inhibiting SGLT2 in the early proximal tubule, which leads to increased urinary glucose excretion by the kidneys, and thus reduce the plasma glucose levels in an insulin-independent manner. Selective sodium glucose co-transporter 2 inhibitors also reduce weight and the amount of visceral adipose tissue (VAT) by caloric elimination, and improve insulin resistance [9–11]. Therefore, SGLT2 inhibitors might be capable of reducing EAT.

Previous pilot studies show that the SGLT2 inhibitors luseogliflozin and ipragliflozin reduce the EAT volume, based on magnetic resonance imaging evaluation [12, 13]. However, it has not been elucidated whether the SGLT2 inhibitor canagliflozin reduces EAT thickness in patients with type 2 diabetes or whether the change in EAT thickness through canagliflozin treatment can be detected with echocardiography. The aim of this study was to clarify whether canagliflozin reduces EAT thickness, based on echocardiography evaluation, in patients with type 2 diabetes.

## Methods

We enrolled 15 patients with type 2 diabetes mellitus and treated them with 100 mg canagliflozin once a day, as a monotherapy or as add-on/combination therapy with existing antidiabetic treatment between May 2016 and December 2016. One meta-analysis showed that the adverse effects of canagliflozin were not influenced by its dosage [14]; however, the effect of canagliflozin on glycated hemoglobin (HbA1c) is dose-dependent [10]. Thus, we fixed the dosage of canagliflozin at 100 mg to exclude the dosage effects of canagliflozin. During the study period, the patients did not alter their use of antidiabetic drugs such as dipeptidyl peptidase-4 inhibitors, α-glucosidase inhibitors, sulfonylureas, biguanides, glinides, thiazolidinediones, and insulin, or other drugs that can affect glucose metabolism such as angiotensin-converting enzyme inhibitors/angiotensin II receptor blockers, statins, diuretics, or β-blockers.

**Fig. 1:**
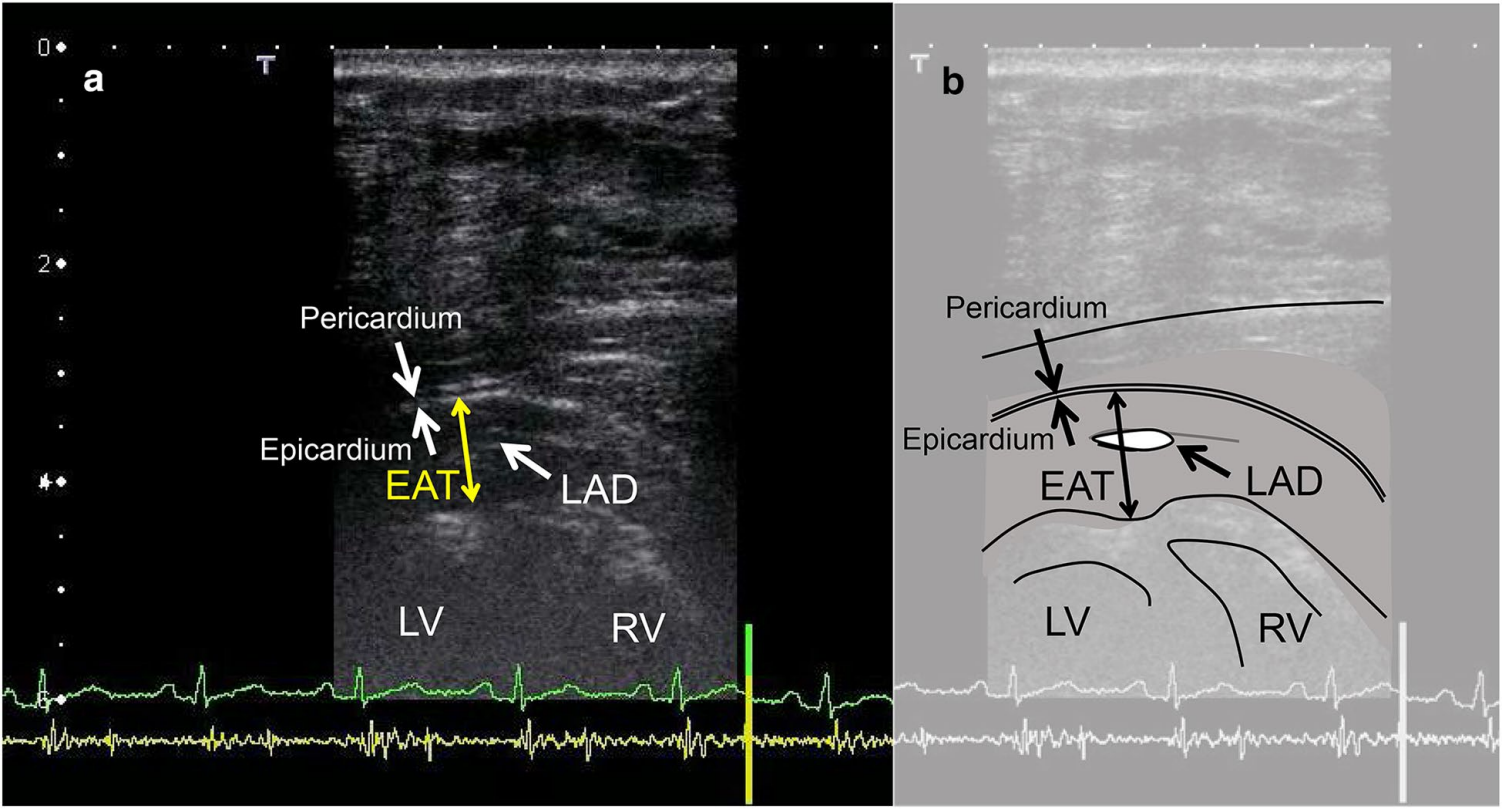
A representative echocardiographic image of epicardial fat tissue. **a** Measurement of epicardial fat thickness using a high-frequency linear probe. **b** Schematic illustration of the echocardiographic image. *EAT* epicardial fat tissue, *LAD* left anterior descending coronary artery, *LV* left ventricle, *RV* right ventricle

Clinical measurements were performed before initiating the treatment and at 3 and 6 months after initiating treatment. Epicardial adipose tissue thickness was evaluated with echocardiography [15]. Echocardiography was performed using commercially available ultrasound machines (Vivid E9, GE Healthcare, Milwaukee, WI, USA or Aplio 500, Toshiba Medical Systems, Tochigi, Japan) with a sector transducer. A high-frequency linear probe (7.5–11 MHz) was used to measure the EAT thickness at the end of systole in the anterior interventricular groove. While assessing EAT thickness, the distal portion of the left anterior descending coronary artery was identified and the probe was carefully rotated until a longitudinal section of the artery was obtained. The EAT thickness was measured as the distance between the outer wall of the myocardium and the visceral layer of the epicardium, perpendicular to the pericardium. Measurements were performed during three cardiac cycles for each parameter, and the mean for each parameter was used for statistical analysis. Interobserver and intraobserver variability were 1.1 ± 1.1 and 0.5 ± 0.5 mm, respectively [15]. All measurements were obtained by a single sonographer who was blinded to the metabolic status of the study participants. A representative echocardiographic image is shown in Fig. 1.

The areas of VAT and subcutaneous adipose tissue (SAT) at the level of the umbilicus were measured using the bioelectrical impedance method with a fat area analyzer (Dual Scan HDS-2000^®^; Omron, Japan). Previous studies have shown a strong correlation between the visceral fat area measured with the fat area analyzer and the visceral fat area measured with computed tomography [16, 17].

Whole body skeletal muscle mass was measured using multifrequency bioelectrical impedance method with a body composition analyzer (Inbody720; Biospace, Seoul, Korea) [18].

The glucose-lowering efficacy of SGLT2 inhibitors was assessed by measuring the HbA1c level before initiating therapy and at 3 and 6 months after initiating therapy, in addition to assessment by using other laboratory data.

The exclusion criteria included active malignancy and current therapy with drugs that affect glucose metabolism, if they were to be altered during the study period.

Informed consent was obtained from all individuals before enrollment. This study was carried out in conformance with the declaration of Helsinki and the study protocol was approved by the Tokushima University Hospital Ethics Committee (Tokushima, Japan; Approval Number, 2549). In accordance with the Japanese governmental ethical guidelines and the Study Protocol (UMIN000021327), monitoring was performed by members of the Clinical Trial Center for Developmental Therapeutics, Tokushima University Hospital (Tokushima, Japan).

## Statistical analysis

For continuous variables, the average was estimated and each value was expressed as the mean ± the standard deviation and categorical parameters were expressed in percentages. Clinical data before initiating therapy with canagliflozin and at 3 months and at 6 months after initiating it were compared using the paired t-test. All statistical analyses were performed using JMP software (version 11; SAS Institute, Cary, NC, USA). Statistical significance was defined as P < 0.05.

## Results

### Clinical characteristics of study patients

Characteristics of the patients enrolled in this study are shown in Table 1. Overall, these patients had moderate diabetes, an HbA1c level of 7.1 ± 0.5%, and a body mass index of 27 ± 5 kg/m^2^.

**Table 1:** Clinical characteristics of patients

Variables	
Number of patients	13
Male, n (%)	5 (38%)
Age, years	62 ± 12
Body mass index, kg/m^2^	27 ± 5
Coronary risk factors	
Dyslipidemia, n (%)	7 (54%)
Hypertension, n (%)	8 (62%)
Current sumoker/past smoker	0/3 (23%)
Coronary artery disease	4 (31%)
History of old myocardial infarction	0 (0%)
History of PCI	3 (23%)
History of CABG	1 (8%)
Anti-diabetic drugs	
DPP-4 inhibitors	5 (38%)
α-Glucosidase inhibitors	1 (8%)
Sulfonylureas	1 (8%)
Biguanides	1 (8%)
Glinides	1 (8%)
Thizoladinediones	0 (0%)
Insulin	2 (15%)
Statins	7 (54%)
Antihyperuricemics	3 (23%)

*PCI* percutaneous coronary intervention, *CABG* coronary artery bypass grafting, *DPP-4* dipeptidyl peptidase-4

Unless indicated otherwise, data are presented as mean ± standard deviation

Over the 6-month period, none of them experienced severe side effects such as hypoglycemic episodes, which would have resulted in a hospital visit or hospitalization. However, two patients were excluded from the analysis because one patient stopped taking canagliflozin owing to generalized fatigue and the other patient was found to have stage IV gastric cancer and discontinued this therapy.

### Effects of SGLT2 inhibitors on blood glucose and HbA1c levels

**Fig. 2:**
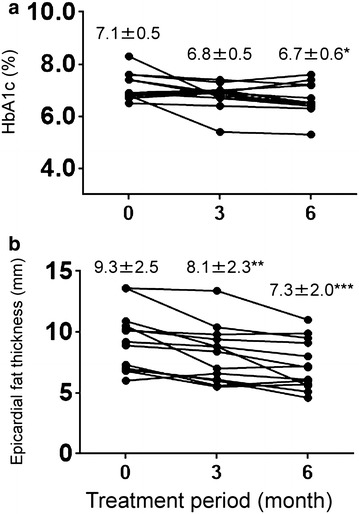
The effects of canagliflozin on the glycated hemoglobin (HbA1c) level and epicardial fat thickness. Values before initiating therapy and after 3 or 6 months are compared. **a** Canagliflozin decreased the HbA1c level at 6 months of therapy. **b** Canagliflozin decreased the epicardial fat tissue thickness at 3 and 6 months of therapy. *P < 0.05, **P < 0.01, and ***P < 0.001, compared to values before initiating therapy

At 6 months of therapy, canagliflozin had significantly decreased the HbA1c level from 7.1 ± 0.5% to 6.7 ± 0.6% (P < 0.05) (Fig. 2).

### Effect of SGLT2 inhibitors on EAT, VAT, SAT, and skeletal muscle weight

Canagliflozin significantly decreased the EAT thickness from 9.3 ± 2.5 to 8.1 ± 2.3 mm at 3 months (P < 0.01) and to 7.3 ± 2.0 mm at 6 months (P < 0.001) (Fig. 2). The VAT significantly decreased from 109 ± 44 to 97 ± 46 cm^2^ at 3 months of therapy (P < 0.05), but was not different at 6 months of therapy. The SAT significantly decreased from 193 ± 71 to 177 ± 81 cm^2^ at 3 months of therapy (P < 0.05), but was not different at 6 months of therapy (Table 2). Canagliflozin therapy did not affect the skeletal muscle weight (Table 2).

**Table 2:** The effects of canagliflozin on anthropometric and laboratory data

Variables	Before treatment	3 month after treatment	6 month after treatment
Body weight, kg	69 ± 13	69 ± 13	67 ± 11*
VAT area, cm^2^	109 ± 44	97 ± 46*	101 ± 47
SAT area, cm^2^	193 ± 71	177 ± 81*	182 ± 82
Skeletal muscle weight, kg/m^2^	15 ± 2	15 ± 2	15 ± 2
Systolic blood pressure, mmHg	131 ± 16	131 ± 20	125 ± 22
Diastolic blood pressure, mmHg	73 ± 11	77 ± 10	72 ± 8
Heart rate, b.p.m	75 ± 11	76 ± 11	76 ± 17
Hematocrit, %	42 ± 5	43 ± 5*	45 ± 6**
Serum creatinine, mg/dL	0.8 ± 0.3	0.9 ± 0.3	0.9 ± 0.3
eGFR, ml/min./1.73 m^2^	65 ± 19	63 ± 20	62 ± 18
Urinary albumin, mg/g ceratinine	37 ± 54	26 ± 27	33 ± 32
LDL-choresterol, mg/dL	106 ± 36	114 ± 42	119 ± 44
HDL-cholesterol, mg/dL	58 ± 21	58 ± 19	60 ± 20
Triglyceride, mg/dL	189 ± 100	160 ± 88	170 ± 121
Uric acid, mg/dL	4.9 ± 1.3	4.2 ± 1.1**	4.2 ± 1.0**

*eGFR* estimated glomerular filtration rate, *LDL* low-density lipoprotein, *HDL* high-density lipoprotein, *VAT* visceral adipose tissue, *SAT * subcutaneous adipose tissue

Data are presented as mean ± standard deviation. * P < 0.05 vs before treatment, ** P < 0.01 vs before treatment

The change in EAT thickness at 6 months was not associated with a change in HbA1c, which indicated that the effect of canagliflozin on reducing EAT is independent of its glucose-lowering effect (Additional file 1: Figure S1). In addition, the change in EAT thickness was not associated with the change in the surface area of VAT or SAT or the change in body weight at 6 months (Additional file 1: Figure S2).

### Effect of SGLT2 inhibitors on laboratory data

There was a significant increase in the hematocrit from 42 ± 5% to 43 ± 5% at 3 months (P < 0.05) and to 45 ± 6% at 6 months (P < 0.01). There was also a significant decrease in the serum uric acid level from 4.9 ± 1.3 to 4.2 ± 1.1 mg/dL at 3 months (P < 0.01), and to 4.2 ± 1.0 mg/dL (P < 0.01) at 6 months of therapy with canagliflozin. However, no effects were observed on the levels of low-density lipoprotein (LDL) cholesterol, high-density lipoprotein (HDL) cholesterol, triglycerides, serum creatinine, estimated glomerular filtration rate, and urinary albumin (Table 2).

## Discussion

We demonstrated that canagliflozin reduces EAT at 3 and 6 months without affecting VAT, SAT, and muscle weight. Epicardial adipose tissue, a lipid storage depot, covers the surface of the heart and surrounds the coronary arteries while also functioning as an endocrine organ secreting hormones and inflammatory cytokines [19]. Epicardial adipose tissue releases proinflammatory and proatherogenic cytokines, including tumor necrosis factor-α, monocyte chemoattractant protein-1, interleukin-6, nerve growth factor, resistin, visfatin, omentin, leptin, plasminogen activator inhibitor-1, and angiotensinogen [19]. These cytokines influence coronary atherogenesis and myocardial function because there is no fibrous fascial layer to impede the diffusion of free fatty acids and adipokines between EAT and the underlying vessel wall, as well as the myocardium [19]. Thus, reducing EAT can help in preventing CAD.

It has been reported that weight loss [20], bariatric surgery [8], exercise [21], or atorvastatin therapy [22, 23] lead to a regression of EAT. A meta-analysis has shown that exercise has little effect on reducing EAT and body weight [8]. On the contrary, diet therapy reduces EAT and body weight, which indicates that caloric elimination has more preferable effects, compared to caloric consumption. Therefore, SGLT2 inhibitors can be good candidates to reduce EAT effectively. Recent studies have shown that luseogliflozin and ipragliflozin treatment for 12 weeks reduced EAT volume, which was evaluated by magnetic resonance imaging evaluation [12, 13].

It has been reported that canagliflozin reduces VAT in 52 weeks [10]. However, the effect of canagliflozin on EAT has not been investigated. In our study, canagliflozin treatment demonstrated a tendency to reduce VAT, but it was not statistically significant in 6 months because of the heterogeneity of the participants. However, canagliflozin treatment reduced EAT by 3 months, which was earlier than we expected. Precise mechanisms underlying these results are unknown. However, EAT has a higher rate of fatty acid intake and secretin than VAT, and functions as a local energy source during times of energy demand. The high metabolic turnover might explain why EAT was more sensitive to SGLT2 treatment, compared to VAT [24].

Epicardial adipose tissue has been evaluated with magnetic resonance imaging or computed tomography (CT) to quantify its thickness and volume [25, 26]. Magnetic resonance imaging and CT are superior to echocardiography for measuring overall EAT volume. Echocardiography is advantageous because it is noninvasive, costs less, does not expose the patient to radiation, and is easier to perform, although it provides only measurements of the regional thickness of EAT. Recent evidence has shown that echocardiographic epicardial fat thickness reflects visceral adiposity rather than general obesity, which is correlated with metabolic syndrome, insulin resistance, CAD, and subclinical atherosclerosis. Thus EAT evaluation with echocardiography is a simple tool for estimating cardiometabolic risk [27–29]. We previously demonstrated that the reliability of our method of evaluating EAT thickness in the anterior interventricular groove to predict the presence of CAD is similar to that of CT scan [15]. A meta-analysis demonstrated that EAT thickness in the anterior interventricular groove is associated with CAD [30]. In addition, we used a linear probe with high frequency (7.5–11 MHz) with high special resolution of 0.1–0.2 mm, which is used clinically to measure the intima-media thickness of the carotid artery. The sector probe that is used for usual cardiac scanning is a lower frequency probe (2–3 MHz) with a low special resolution of 0.5 mm, which might be insufficient to evaluate EAT thickness. Thus, by using this noninvasive and highly sensitive method, we monitored EAT periodically during the therapy and detected the fine change in EAT thickness after canagliflozin treatment.

We showed that canagliflozin decreased the level of uric acid. The mechanism by which SGLT2 inhibitors reduce the level of uric acid has not been fully established. However, the facilitative glucose transporter 9 isoform 2 (SLC2A9b) may be involved: it is expressed at the apical membrane of the renal tubular cells and exchanges glucose for uric acid [31]. Higher glucose concentrations in urine, which are attributable to canagliflozin treatment, could increase the exchange of uric acid in the apical membrane of tubular cells through the transporter, increase the release of uric acid from blood into the urine, and thereby reduce the serum uric acid levels. This potential mechanism has been supported by the evidence of trans-stimulation of uric acid efflux with high glucose concentrations in *Xenopus* oocytes expressing SLC2A9b [32].

The present study had several limitations. First, the study is a single-arm observational study with a small sample size. Thus, the study contains a patient selection bias and less statistical power due to the patients’ heterogeneity. Second, the observational period was relatively short. Hence, the long-term effects of SGLT2 inhibitors could not be analyzed. Third, we could not exclude the effects of concomitant drugs that can affect glucose metabolism, even though the patients’ drugs were not changed during the study period. Fourth, because of a methodological issue, we evaluated only EAT thickness and did not study the effect of canagliflozin on EAT volume. Randomized, large, clinical cohort studies with a longer observation period are needed to evaluate further the findings of this study.

## Conclusion

Canagliflozin treatment for 6 months reduced EAT thickness, as evaluated by echocardiography, in patients with type 2 diabetes mellitus, independent of its effect on lowering blood glucose, which suggests that canagliflozin may have an effect in preventing cardiovascular events in these patients.

## Additional file

**Additional file 1: Figure S1.** The change in HbA1c at 6 months is not associated with changes in EAT thickness, VAT area, or SAT area at 6 months. EAT, epicardial adipose tissue; HbA1c, glycated hemoglobin; SAT, subcutaneous adipose tissue; VAT, visceral adipose tissue. **Figure S2.** The change in EAT at 6 months is not associated with changes in VAT area, SAT area, or body weight at 6 months. EAT, epicardial adipose tissue; SAT, subcutaneous adipose tissue; VAT, visceral adipose tissue.

## Abbreviations

CT: computed tomography
EAT: epicardial adipose tissue
CAD: coronary artery disease
HbA1c: hemoglobin
A1c HDL: high-density lipoprotein
LDL: low-density lipoprotein
SGLT2: selective sodium glucose co-transporter 2
SAT: subcutaneous adipose tissue
VAT: visceral adipose tissue
