# Supplementary material for: Canagliflozin reduces epicardial fat in patients with type 2 diabetes mellitus
Source: Diabetol Metab Syndr. 2017 Oct 4;9:78. doi: 10.1186/s13098-017-0275-4 (PMC5628447; doi:10.1186/s13098-017-0275-4)
Supplement: Supplementary file 1 — Additional file 1: Figure S1. The change in HbA1c at 6 months is not associated with changes in EAT thickness, VAT area, or SAT area at 6 months. EAT, epicardial adipose tissue; HbA1c, glycated hemoglobin; SAT, subcutaneous adipose tissue; VAT, visceral adipose tissue. Figure S2. The change in EAT at 6 months is not associated with changes in VAT area, SAT area, or body weight at 6 months. EAT, epicardial adipose tissue; SAT, subcutaneous adipose tissue; VAT, visceral adipose tissue. [file 13098_2017_275_MOESM1_ESM.pptx]

## Slide 1
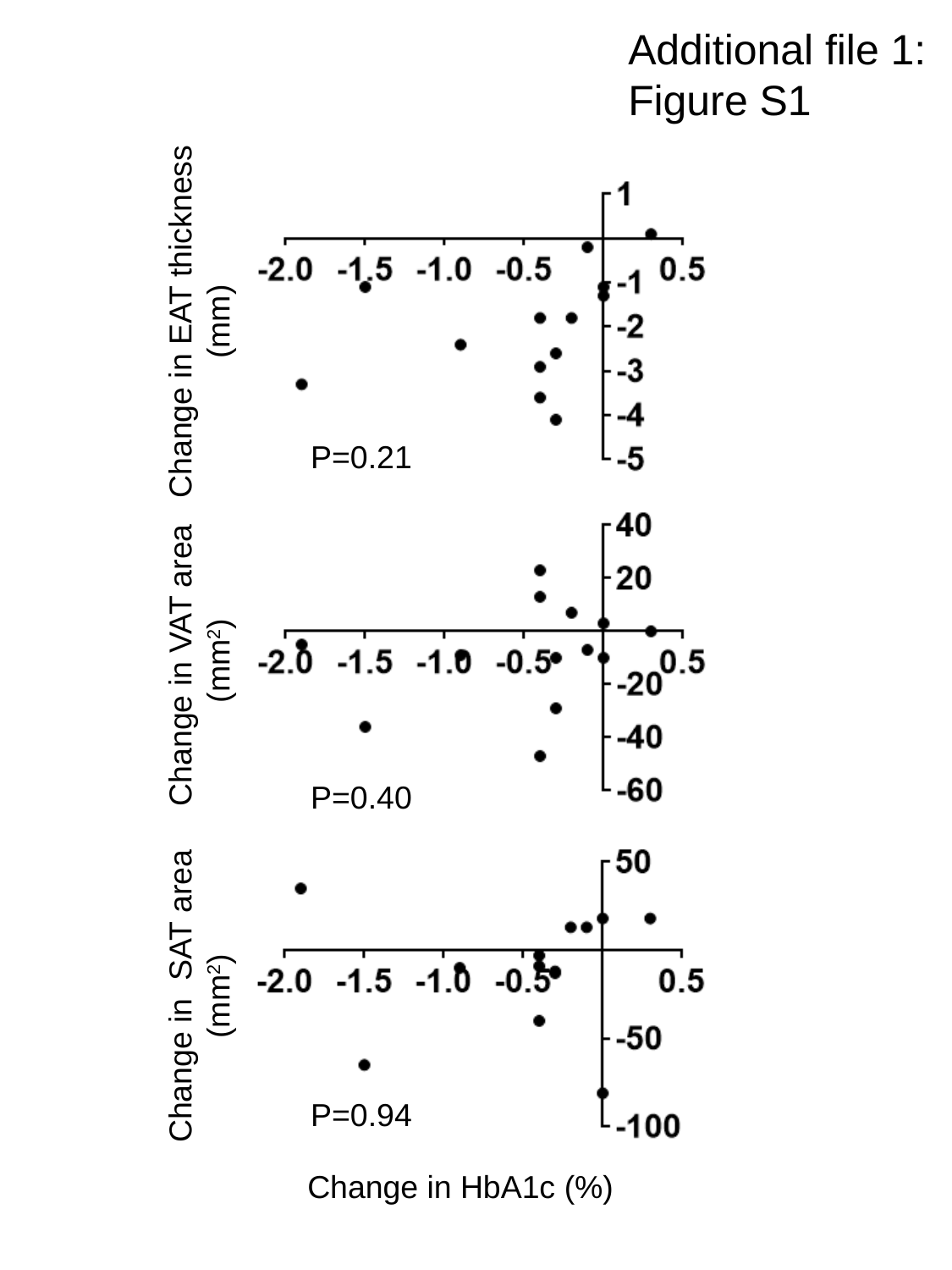

Additional file 1: Figure S1
Change in EAT thickness
(mm)
P=0.21
Change in VAT area
 (mm2)
P=0.40
Change in SAT area
(mm2)
P=0.94
Change in HbA1c (%)

## Slide 2
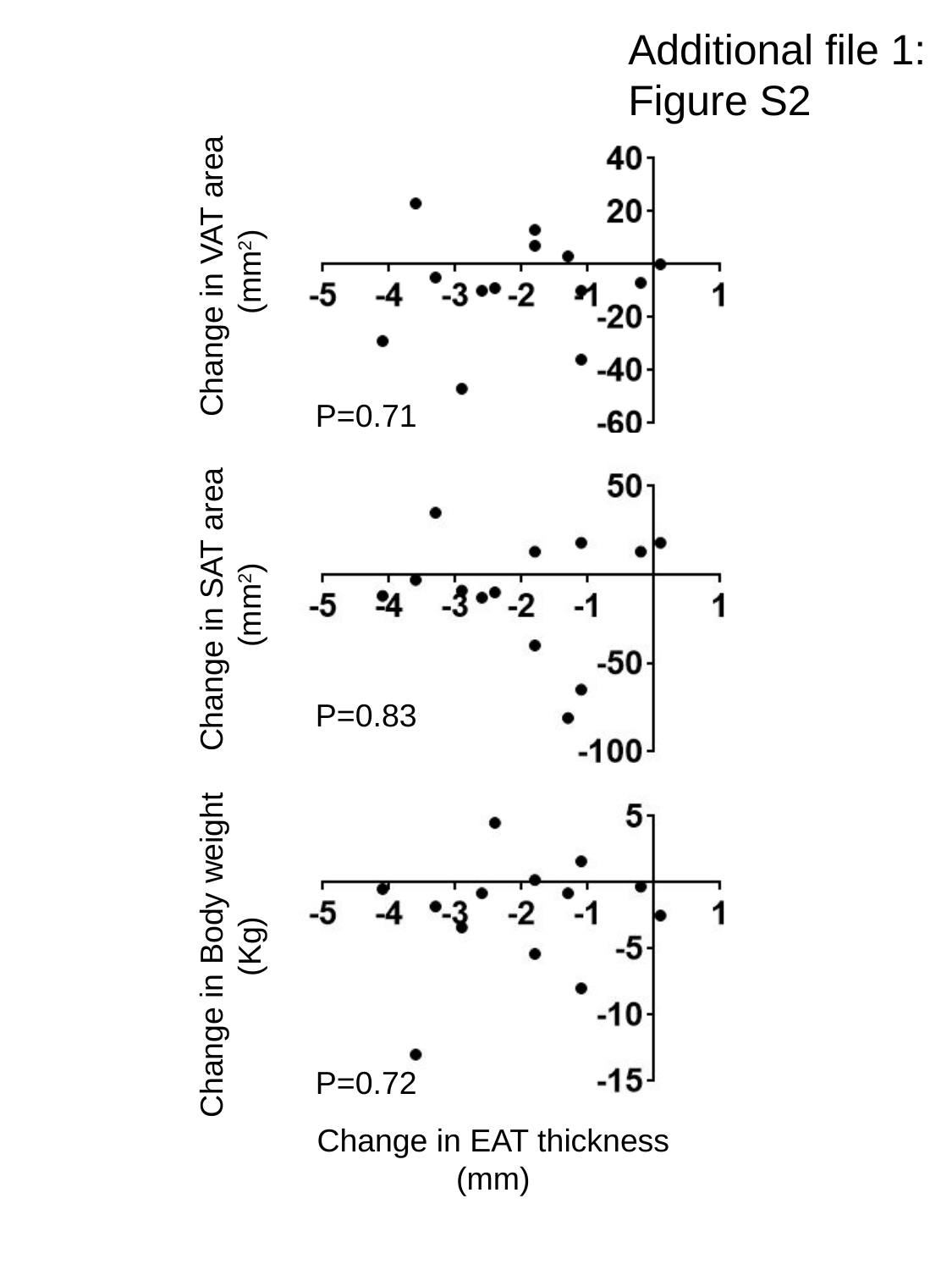

Additional file 1: Figure S2
Change in VAT area
 (mm2)
P=0.71
Change in SAT area
 (mm2)
P=0.83
Change in Body weight
 (Kg)
P=0.72
Change in EAT thickness
(mm)
